# Hopomics: *Humulus lupulus* Brewing Cultivars Classification Based on LC-MS Profiling and Nested Feature Selection

**DOI:** 10.3390/metabo12100945

**Published:** 2022-10-05

**Authors:** Yuriy Andreevich Ikhalaynen, Ivan Victorovich Plyushchenko, Igor Alexandrovich Rodin

**Affiliations:** 1 Chemistry Department, Lomonosov Moscow State University, 119991 Moscow, Russia; 2 Department of Epidemiology and Evidence-Based Medicine, Sechenov First Moscow State Medical University, 119435 Moscow, Russia

**Keywords:** *Humulus lupulus*, metabolomics, untargeted profiling, machine learning

## Abstract

Omics approaches in plant analysis find many different applications, from classification to new bioactive compounds discovery. Metabolomics seems to be one of the most informative ways of describing plants’ phenotypes, since commonly used methods such as liquid chromatography–mass spectrometry (LC-MS) and nuclear magnetic resonance spectroscopy (NMR) could provide a huge amount of information about samples. However, due to high efficiency, many disadvantages arise with the complexity of the experimental design. In the present work, we demonstrate an untargeted metabolomics pipeline with the example of a *Humulus lupulus* classification task. LC-MS profiling of brewing cultivars samples was carried out as a starting point. Hierarchical cluster analysis (HCA)-based classification in combination with nested feature selection was provided for sample discrimination and marker compounds discovery. Obtained metabolome-based classification showed an expected difference compared to genetic-based classification data. Nine compounds were found to have the biggest classification power during nested feature selection. Using database search and molecular network construction, five of them were identified as known hops bitter compounds.

## 1. Introduction

*Humulus lupulus* is one of the species representing the genus *Humulus* in the family *Cannabaceae*. It is a perennial, herbaceous, climbing liana that grows in a temperate climate. This plant left a remarkable trace in different countries’ cultures, mainly as a medicinal plant. As well as a multifunctional beer drinks component, it acts both as a preservative and one of the main flavoring properties of the drink’s sources. Nowadays, the brewing industry remains the main consumer of hops. Volume of annually harvested hops is estimated at hundreds of thousands of tons [1]. For the needs of brewing, female flowers are required, which have a cone-shaped form and glands on their surface. The last secrete the bulk of the secondary metabolites that compose the plant flavor profile. Breeding techniques development and wide distribution of hops around the world let us have more than a hundred varieties cultivated nowadays [2], some of which were bred already in the 21st century [3,4]. In addition, wild hops are also considered as a raw material for brewing [5,6]. The multiple techniques used in beer hopping as well as the wide range of hop varieties cultivated today [7] make it possible to produce beverages with a rich palette of flavors and aromas [8].

The lack of genealogical information about the individual species’ origin is formed due to the absence of systematizing information. The protocols about the derived cultivars and their initial stage of breeding development are unavailable due to commercial secrecy. All this together makes it difficult to systematize used varieties. To solve this problem, there are many approaches that can be divided into two groups: genetic and phenotypic. The development of genetic techniques, including whole genome sequencing, has opened opportunities for the taxonomic classification of various organisms, both based on the complete DNA sequence analysis [9] and selecting individual, most informative regions (EST-SSR (expressed sequence tag-simple sequence repeat)), after obtaining information about the reference sample genome [10]. Due to the peculiarities of the plant genome [11], the last approach is more popular and applicable to this type of organism. Among the diversity of ways to describe the phenotypic variation of plants, it can be distinguished: classical morphological classification [5], transcriptome [12], proteome [13], and metabolome analysis. Despite the fact that all the methods above solve different problems, the metabolomic approach is especially suitable for plant analysis and is much more informative. The competent use of modern instrumental analysis methods makes it possible to conduct both qualitative and (semi)quantitative studies. In addition, since the plant metabolome is responsible for biological activity and flavor profile, metabolomic analysis is the most valid technique for studying plants such as hops.

Secondary metabolites of hops represent a wide range of compounds. If the taste and the scent of a plant (or products based on it) are considered, as well as its biological activity, several of the most significant chemical classes should be underlined: alpha and beta acids, flavonoids, prenylflavonoids, as well as a vast group of volatile compounds. At the moment, many “bitter” components [8,14,15] of hops have been described; the main part of their composition is alpha acids (humulone and its derivatives), beta acids (lupulone and its derivatives) and prenylargenins (xanthohumol, etc.). For the main compounds of these classes different types of biological activity were shown: antimicrobial, antioxidant and antitumor [16,17]. The most suitable and informative tool for separation and detection of compounds mentioned above is LC-MS in reversed-phase mode, especially when untargeted metabolomics study is planned. Despite many studies devoted to establishing the composition of hops, including isolation and subsequent structure identification of compounds, only a limited number of compounds are presented in publicly available databases such as KnapSack [18]. That makes it difficult to analyze untargeted metabolomics data, because it is necessary to deal with unsystematized information from literary sources.

Despite all the advantages of metabolomics-based plant analysis, there are some obstacles associated with both the approach itself and the study of specific objects of the plant world: the impossibility of complete coverage using only one method/type of analysis, the need to deal with high dimensional data, the complexity that arises when taking into account all external factors influencing the formation of the natural sample, which can complicate classification problems, as well as technical factors of used analytical methods.

However, most of these problems disappear when the object of study is a human cultivated plant, where each sample will contain many input data and, more importantly, to sample collection with biological replicates is notably easier. In this regard, it is worth noting the possibility of using such objects to optimize approaches for data preprocessing and statistical analysis with the opportunity of their further translation to more complex subjects. Mentioned specifics of cultivated plants lead us to choose *Humulus lupulus* as an object of our study.

Thus, we state that the objective of our study was to perform LC-MS-based untargeted metabolomics study on *Humulus lupulus* brewing cultivars for group-markers compounds search. Comparison of two types of classification (metabolome- and genome-based) was also conducted and evaluated. As studied species is a convenient object for untargeted research, so the developed experimental design and data processing scheme could be used to address other related issues.

## 2. Results and Discussion

### 2.1. Study’s Background and Sample Collection

Despite the confidence level of plant classification based on phylogenetic methods, specifics of connections between phenotype and genotype could lead to significant differences in results of different chemotaxonomy and classic morphological methods applied to the same object. From the gene-based approaches side, this could be induced by the absence of a predictive marker in the chosen set (for methods such as EST-SSR) or the big influence of a single gene expression on overall plant phenotype, and as a result, on its metabolome [19,20]. For human cultivated species, both genetic and metabolomic approaches are important: it is necessary to estimate connections between cultivars on both genetic and small organic molecules’ composition levels. Considering all mentioned details, *Humulus lupulus* was chosen as our study object, for proving the hypothesis that there is no clear correlation between genome- and metabolome-based classification for plants. Influence of the region of growth was also going to be estimated. The collection was formed (see Section 3.2) based on the sample’s region of growth and genetic origin, which was obtained from the study on hops EST-SSR-based classification [10]. A total of 18 cultivars were analyzed in the present study, each was represented by three biological repeats (2019–2020 harvests). Samples were divided into three groups based on their geographic origin: Central Europe, North America and Oceania (six samples in each); in two groups by genetic classification [10]: North American (6) and European germplasm (12); and also in two groups according to plant usage in beer flavoring: aroma (10) and dual use (8). Detailed information about selected cultivars is provided in Table S1.

### 2.2. Data Pre-Treatment

**Figure 1:**
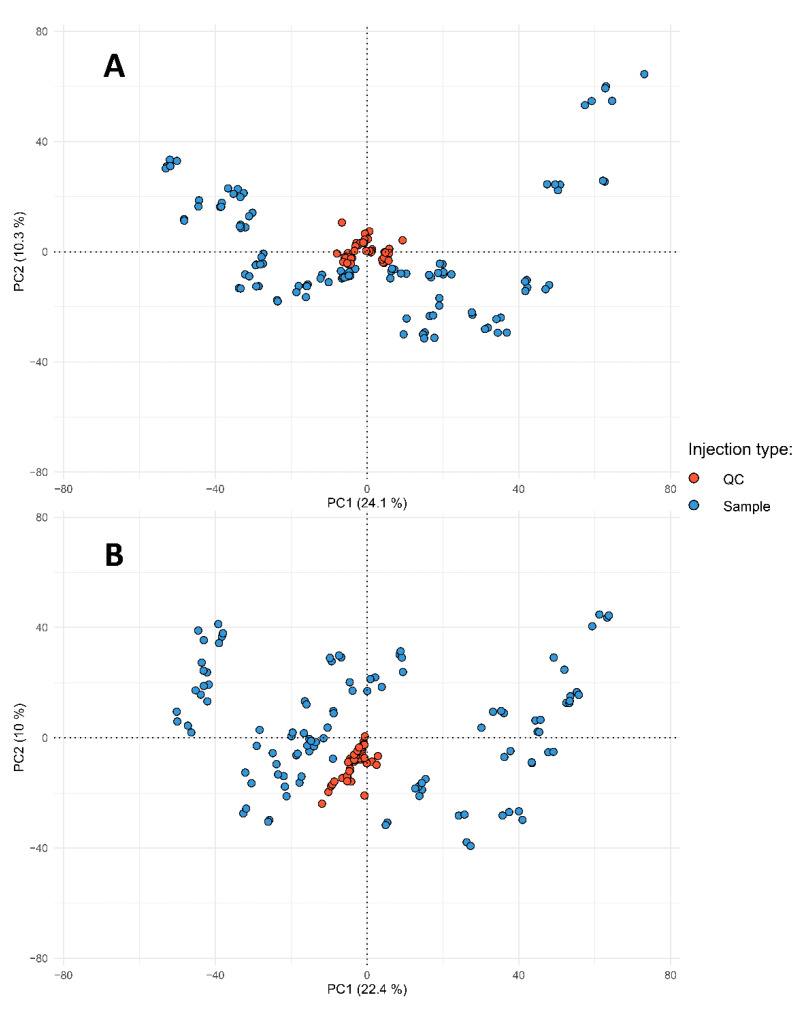
Quality assurance projections of study samples on principal components surface. “**A**” corresponds to the positive ion’s signals table, “**B**”—negative.

Usage of LC-MS instruments provides a huge, informative dataset for metabolomics research, but the complexity of both LC and MS instruments together with specifics of the large-scale analysis brings many issues, such as batch and matrix effects [21], which could affect results of the following statistical analysis. Most of the negative effects could be reduced by the development of experimental design, including experimental sample randomization [22], usage of quality control (QC) and blank samples [23] and careful data pre-processing steps before results interpretation. There are various workflows and tools designed for LC-MS data pre-treatment [24,25,26,27], and most of them are based on R-programming language and corresponding packages. The pipeline of the present study was based on ”*Omics Untargeted Key Script*” [28] (computational details in Section 3.5, experimental design and LC-MS instrumentation—3.3, 3.4). The missing value filling step was done by Random Forest model-based imputation strategy described in [29], as this approach works well on samples with large similarity, which positively affects model construction time. Principal component coordinates were computed for QC and individual samples for quality assurance purposes. As displayed in Figure 1, all QC samples are placed in the center in case of both positive and negative ion signals, which indicates properly prepared QC samples and well-constructed experimental sequences.

Before the signal correction step, signal reproducibility was checked by RSD [30] calculation between QC samples—88% (3127) and 90% (3023) of signals had RSD less than 30% for negative and positive polarity, respectively. The XGBoost model was utilized for signal correction as described in Section 3.5. After the correction step, RSD value among QC samples was checked again as mentioned above and, as corrected peak areas had good reproducibility between QC samples, no features have been filtered. All steps mentioned above allowed us to reduce the influence of the unwanted technical variations and perform further classification and feature selection tasks correctly.

### 2.3. Metabolome-Based Classification

A large amount of data obtained during metabolomic profiling (more than 3000 peaks in the present study) leads to confusion in further results. Dimensionality reduction techniques can be performed in two ways: feature selection by statistical tests or machine learning methods to eliminate statistically non-informative variables and feature extraction by principal component analysis (PCA) or other algorithms in order to transform the whole feature space into new variables. PCA and other similar data representation techniques [31] are also often used in the first steps of LC-MS experimental data treatment for exploratory purposes, such as quality assurance [23]. In some cases, it is even possible to cluster sample dots for group labels on a score plot by visual analysis of the first two or three principal components spaces [32,33]. In the present study, a combination of two unsupervised methods was utilized (see Section 3.6 for more details). PCA was employed for multivariate projection and HCA was performed both for distance calculation between sample dots on the score plot and assignment of the cluster group. The procedure of receiving a new sample group label should be considered as one of the primary aims of the present study, due to the lack of solid confidence in cultivars labels based on genetic data, despite its relevance compared to information about the geographic origin or cultivar’s aroma characterization [34]. In a similar way, a combination of different algorithms (both univariate and multivariate) was applied for feature selection and dimensionality reduction.

**Figure 2:**
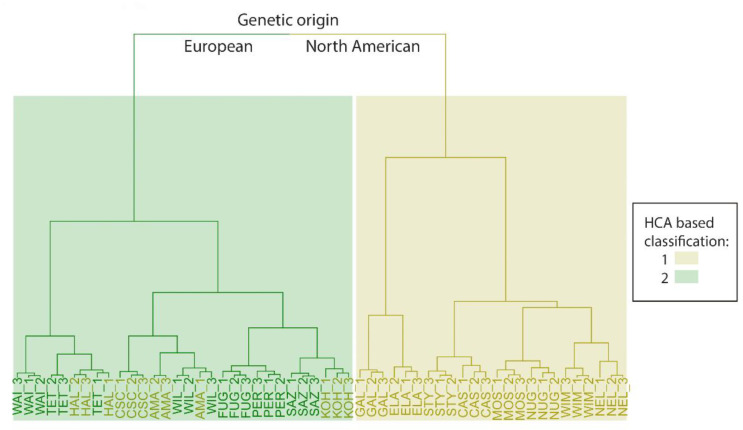
The results of PC-based HCA. The genetic origin of the samples is presented as the color of leaves and sample labels. Newly obtained groups are represented as a background color. Definitions of sample labels are provided in the Table S1.

**Figure 3:**
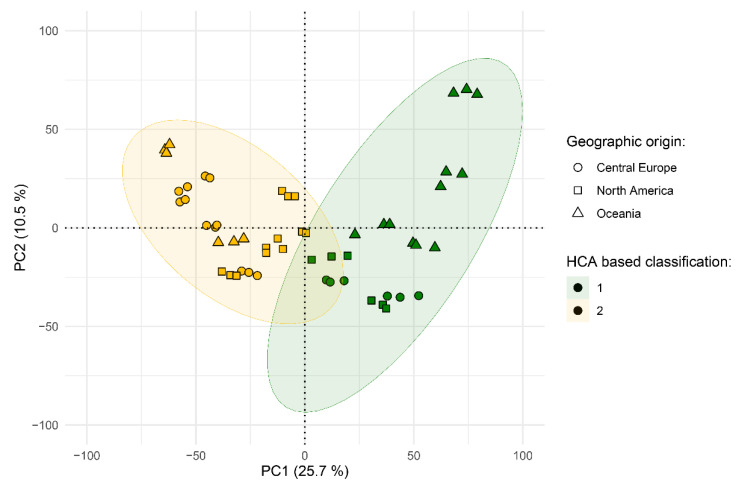
Projection of study samples on principal components surface. Newly assigned groups are demonstrated by color, geographic origin is represented by points shape.

Thus, class labels were received from the combination of both information sources: HCA results (Section 3.6, Figure 2, Table 1) and the geographic origin of the samples, and PCA was used to describe the distribution of experimental samples in the high-dimensional feature space (Figure 3).

As shown, HCA assigned cluster membership similar to their visually observable location. Furthermore, for each sample, biological repeats are grouped together, so it can be concluded that there is no significant difference between harvests. Compared to genetic classification results from [10], all “European germplasm” origin samples were assigned to the same cluster with four more cultivars from the “North American germplasm” group: Hallertau Mittelfruh, Amarillo, Cascade and Kohatu, which are reported to contain relatively low (3–7%) alpha-acids in their composition. Such results prove the hypothesis that there is no clear correlation between plants’ genome and metabolome. Genomic research, unfortunately, could not tell us about expression level of genes related to enzymes responsible for synthesis of the small organic molecules in the plant. Additionally, it is typical for plants to contain wide non-coding or some kind of regulatory regions, which do not have a direct influence on the resulting phenotype. Additionally, obtained sample labels, in most cases, match accepted cultivars classification [34] by aroma and flavor, even in conditions when volatile compounds, which primarily affect *Humulus lupulus* taste and odor characteristics, have not been the aim of our work. Another interesting task is a comparison between cultivar genetic and geographic origins. As an example, studies [35,36] were focused on untargeted metabolomics analysis for cultivar and geographic origin discriminations of olive oil and hazelnut (*Corylus avellana* L.) and significant overlapping between marker compounds for those two levels of classification was not reported. As the plot above shows, only some cultivars from the Oceania region seem to be distancing from the studied dataset (see Figure S6 for plot with marked sample names). Internationally standardized harvest conditions of studied species, as well as the major influence of genotype, could explain such a situation. As the region of growth does not have a significant influence on our sample, in the next step, most discriminative marker compounds for our metabolome-based classification, which is based on HCA results, were derived using a rational cut-off approach in further statistical analysis.

Rational cut-off is one of the most frequently used techniques in chemometrics for feature selection. It is usually based on the simultaneous application of statistical tests combination to a studied dataset with different rigors and natures, which leads to the selection of only variables that satisfy a list of statistical conditions (cut-off). In the present work for feature selection, two classical mean comparison tests with different rigor levels were utilized: moderated t-test (as one of the most rigorous test for comparing means due to shrink deviation of variables) and fold change calculation, and also partial least squares (PLS) classification model computation as an “orthogonal” supervised multivariate method. A moderated t-test was implemented by Limma package [37] with Benjamini−Hochberg *p*-value adjustment method for multiple comparisons. Those calculations were implemented in a nested-like feature selection scheme (Section 3.6) [38,39] to avoid negative effects associated with small dataset size. Cross-validation based on 10-fold splitting (outer loop, Section 3.6) was performed in order to avoid selection bias effect [40] and to provide multiple estimations of classification ability. Classification accuracy was tested with a generalized linear model (GLM) on each outer-fold, which showed maximum classification accuracy for each of 10 dataset splits. Bootstrapping on each inner fold was performed to reduce the influence of the small dataset size. Obtained bootstrapped datasets were further proceeded through a rational cut-off step.

Sixty-two signals met the criteria established for maximum prediction power. Finally, the descriptive ability of selected metabolites was checked by providing HCA as well as the distribution of sample dots on the space of the first two principal components (Supplementary material, Figures S4 and S5). Misclassifications were not observed in the case of cluster analysis and the visual separation of groups on the PCA plot was increased.

It should be noted before structure elucidation that signal annotation was not performed at the preprocessing stages. As a result, among 62 derived signals (33 for positively charged ions and 29 for negative), 19 groups of signals were clustered by retention time. Each group contained expected a de- or protonated molecular ion signal, isotope and adduct signals, as well as smaller *m/z* values, which could be assigned to individual compounds or to ions formed during in-source fragmentation. The signal’s type was investigated during subsequent analysis of the fragmentation spectra.

### 2.4. Identification of Marker Compounds

The correct way of unknown identification in complex mixtures such as plant extracts is to isolate compounds in their individual state and to provide multi-methods analysis for structure verification, as it was previously performed for studied plants in works [41,42,43,44]. However, as target compounds could occur in analyzed samples at extremely low concentrations, their isolation becomes very time-consuming. In that case, information obtained from LC-MS experiments: relative retention time, accurate mass, isotope distribution pattern and fragmentation spectra can help to identify a putative structure of a target compound with variable levels of confidence [45]. Furthermore, as not every plant species is studied enough and literature data is not systematized, not much information could be found in public mass-spectra databases compared to a situation with mammalian metabolites. So, the only way to identify minor plant components is to use such databases and resources as KNaPSAcK [18] or GNPS [46] in combination with tools for fragmentation spectra prediction [47,48] or molecular networking [49]. Such a pipeline allows us to find connections with similar compounds if the target one is not presented in a database. After filtration inside each peak group, only 11 pairs of signals were left (for positive and negative ions), apparently related to protonated and deprotonated molecules. The molecular formula was predicted using the Shimadzu Formula Predictor tool for each signal. Molecular formulas, prediction errors in ppm, as well as VIP (variable importance in projection) from the PLS model and fold change values are provided in Table 2.

Concentration of all of the derived markers are greater in group 1 (Table 1) as the fold change value is greater than 0. As will be discussed below, most of the identified compounds belong to the alpha-acids class. Differences in the abundance between classes correlate with the results of the classification discussed above in Section 2.3: all cultivars in group 1 are reported to have a high concentration of alpha-acids. Obtained formulas were searched within the KNaPSAcK metabolite library and articles associated with *Humulus* profiling. After that, fragmentation spectra were obtained and processed via the GNPS library search tool (see Section 3.7). To add one more verification level, some major compounds (Xanthohumol, 8-prenylnargenin, lupulone, etc.) were identified on obtained chromatograms to compare relative retention and fragment ions of the marker compounds with data from [50]. Compounds with the same fragmentation spectra as xanthohumol were found in GNPS, but low retention time (10.46 min) and abundance of the peak with the same *m/z* at the later part of the chromatogram allows us to suspect this as isoxanthohumol. Cohumulone and humulone were also identified by the GNPS library and its relative retention matches information from the source mentioned above. Other markers’ relative retention times and fragments were matched to posthumulone and prehumolone from the same article [50]. To add one more verification step, a molecular network between target and major compounds of *Humulus lupulus* has been constructed. To obtain the major plant’s compounds fragmentation spectra, data acquisition in DDA (data-dependent acquisition) mode was provided. Obtained spectra, total ion current (TIC) and extracted ion (XIC) chromatograms and informative parts of the molecular network are presented in Supplementary Materials (Figures S1–S7). There are two informative clusters describing connections between humulone derivatives and between xanthohumol and an unknown compound related to the deprotonated ion with *m/z* 399.1443. Analysis of neutral losses in humulone derivatives spectra confirms all suspected structures. The connection between xanthohumol and the unknown compound is explained by the presence of ions with *m/z* 119.048, 93.034 and 65.003, which are supposedly corresponding to the 4-ethylphenol fragment. Other fragments and their neutral losses are not matched, so it is the only information that can be obtained about the structure. To sum up, five marker signals were identified as known hops’ bitter components and our findings are consistent with previous reports. Three other signals with fold change values close to 1 have not been annotated properly. Thus, the list of marker substances was proposed along with classification performance statistics (Table 2). Suggested markers may be used for *Humulus lupulus* material quality control as well as intragenus discrimination studies.

## 3. Materials and Methods

### 3.1. Chemicals and Reagents

For sample preparation purposes, HPLC-grade methanol (Panreac, Barcelona, Castellar del Vallès, Spain), syringe PET-membrane filters (0.45 μm, Chromafil, Düren, Germany), ultrasonic bath (Saphir, Moscow, Russia) and household coffee-mill (Bosch, Gerlingen, Germany) were used. The mobile phase was prepared using HPLC-UV-grade acetonitrile (Panreac, Barcelona; Castellar del Vallès, Spain), MS-grade formic acid (Fluka, Seattle, DA, USA) and water, which was prepared by Milli-Q deionization system (Millipore, Burlington, MA, USA).

### 3.2. Sample Collection and Preparation

Hop pellets of different cultivars imported by Beervingem (Russia) were purchased at a local brewing store. All samples were stored in hermetic packages at low temperatures (3–5 °C) before and after the homogenization step. As the study object was presented in pellets, for which production out of hope cones technology is unified, there should be no significant loss of its content [51] in this step.

In the sample preparation step, typical workflows for *Humulus lupulus* [14,15,52] were used and performed before each experimental batch, with the exception of homogenization and weighing. A 15 mL tube with 300 mg of homogenized pellets was filled with 10 mL of methanol and then placed in the ultrasonic bath for 1 h (at 25 °C) for extraction. The obtained extract was syringed through a PET-membrane filter and then diluted 10 times with methanol before injection into the LC-MS system. Signal correction procedure and quality control samples were used to eliminate the impact of the batch effect. For QC samples preparation, an equal mixture of all study samples was treated in all steps provided above. The obtained stock solution was stored at low temperature and was diluted the same as the samples.

### 3.3. Data Acquisition

An LCMS-IT-TOF (Shimadzu, Kyoto, Japan) system, equipped with two Nexera LC-20 AD XR pumps and SIL-20 AC XR autosampler was used for untargeted profiling. Chromatographic separation was performed on reversed-phase column Acclaim C18 3 µm (2.1 × 150 mm, Dionex, Sunnyvale, CA, USA) in gradient elution mode with acetonitrile (A) and 0.1% aqueous formic acid (B) as a mobile phase at 0.35 mL/min. Gradient parameters, modified from the work [53] were as follows: 0–5 min, 30% A; 5–15 min, 30–70% A; 15–32 min, 70–95% A; 32–47 min, 95% A; 47–50 min, 95–30% A; 50–55 min, 30% A. Injection volume was 5 µL, column oven temperature—25 °C and autosampler temperature—4 °C.

Hybrid mass-spectrometer consisting of the ion trap and time-of-flight instruments equipped with an electrospray ionization source was used as a detection system. Data were acquired in both positive and negative polarities on scan (MS^1^) detection mode in conditions listed below: interface voltage—4.5 kV for positive and −3.5 kV for negative charged ions transition; CDL and heat block temperature—200 °C; nebulizing gas flow—1.5 mL/min; cooling gas pressure—100 kPa. Mass-spectrometer was operated at 120–850 Da range, repeated three times during the 300 ms period with ion trap accumulation time set to 30 ms.

Orbitrap Exploris 120 mass-spectrometer combined with Vanquish UHPLC system (Thermo Fisher, Waltham, MA, USA) with the same column and gradient elution program as mentioned above were used for fragmentation spectra acquisition in DDA mode for identification purposes, which includes molecular networks construction. Heated ESI source parameters were as follows: Positive (or Negative) ionization, Ion transfer voltage—3500 (2500) V, Sheath/Aux/Sweep Gas—50/10/1 Arb, Ion transfer temperature—325 °C, Vaporizer temperature—350 °C. Scan Parameters: Resolution—30,000 (for both MS^1^ and MS^2^ spectra), scan range—120–800 *m/z*, RF lens—70%, number of microscans—3, data type—centroid. HCD fragmentation parameters: isolation window—1 *m/z*, isolation offset—0.25 *m/z*, collision energy—15, 30, 45, 60 V, data acquisition mode—ddMS2 for major compounds spectra accumulation and target list-based fragmentation for markers.

### 3.4. LC-MS Profiling

**Figure 4:**
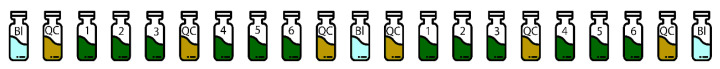
Example of batch sequence used in the experiment. Blank samples are marked as “Bl”.

In order to avoid sample selection bias, randomization of the experimental sequence was performed before LC-MS profiling. The resulting batches were filled with blank- and QC-sampls injections (three and five per batch, respectively) for system stability check, and six chosen samples were analyzed twice per batch (total nine batches were acquired), as shown in Figure 4. Each hop species was analyzed in two technical replicates.

System was cleaned to reduce influence of contamination before and after each batch (chromatographic column with acetonitrile and mass-spectrometer’s ion source, sample cones and skimmer with a mixture of water and isopropyl alcohol).

### 3.5. Computation and Software

The total of 154 raw files (samples and QC data), that were obtained after the experimental step, were then converted to mzXML format using msConvert [54] tool. Peak peaking and subsequent alignment of chromatograms were performed by the xcms [55] R package with parameters that were optimized by IPO [56] in order to obtain a peak table (samples by row, features—columns). Results of the optimization and peak tables for each step are available at the Github repository. Obtained peak tables for each polarity were subjected to the missing value imputation procedure using the missForest [29] package. A QC-based signal correction was performed by the XGBoost model as described in [28], and corrected peak area was calculated by the following equation:

S_corrected_ = S_observed_/S_predicted_ × 1000

After the correction step, features were filtered by their reproducibility in QC samples, and as a result, signals with relative standard deviation (RSD) among QC samples more than 30% were removed. In the next step, technical repeats were averaged and then all data tables were centered and scaled. The final peak table was generated by binding negative and positive polarity corresponding tables by columns.

All dendrograms were generated using Ward.D2 linkage algorithm based on the Manhattan distance between samples. Data matrix was scaled and centered prior PCA in all cases.

Basic operations and tables processing were performed by dplyr, data.table and stringr packages. Data visualization and projection (PCA, HCA) were implemented by FactoMineR and factoextra packages. Figures were created using ggplot2 library.

### 3.6. Semi-Supervised Classification and Feature Selection

**Figure 5:**
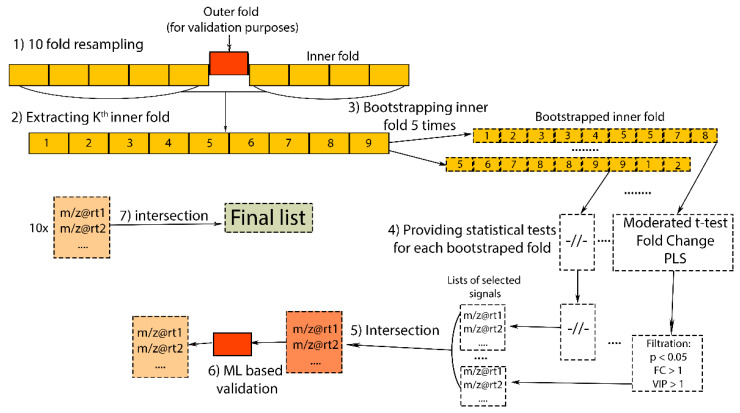
Scheme of implemented nested feature selection algorithm.

In the first step, the number of data dimensions was reduced by computing principal components. Then, in the space of the first 10 principal components, our samples’ coordinates (scores matrix) were extracted, and HCA was computed for clustering samples in two groups (metabolome-based classification). Obtained cluster membership was used to perform subsequent statistical analysis. Nested feature selection was implemented to determine a subset of significant variables, the algorithm is shown in Figure 5. 

Then, 10-fold cross validation splitting was performed on a full experimental dataset (54 samples) as an outer loop. Then, each of all remaining nine inner folds were bootstrapped five times. So, we can validate feature subsets on 10 independent datasets. Feature significance was evaluated and selected by three statistical criteria: adjusted *p*-value from moderated t-test < 0.05, fold change value > 1, and VIP value from PLS model is greater than 1. Then we intersected the results of rational cut-off between resampled data within each bootstrapped inner fold, and, finally, between the overall outer loop. Before final intersection, each signal’s list was validated using a GLM with outer fold for test purposes. The Limma [37] R package was used for adjusted *p*-value and fold change calculations with following parameters: least squares model as fitting method and Benjamini and Hochberg method for *p*-value adjustment. The PLS model was constructed using ropls package [57] with 10-fold cross validation and 100 random permutations for tuning hyperparameters and model evaluation, respectively. GLM for final validation in the outer loop was constructed using the caret [40] package. More detailed information about computations is provided on Github repository.

Before the identification step, isotopic and adduct peaks were removed from the obtained list by manual examination of mass-spectra. For identification of the in-source fragmentation signals, MS^2^ spectra were obtained for each signal as described in the next part (Section 3.7). If peaks were presented in both scan and fragmentation spectra from the ion with greater *m/z* value with the same retention time, this was considered as a molecular ion, but the signal with smaller m/z is assumed as an in-source fragmentation product.

### 3.7. Signal Annotation and Molecular Networks Construction

Molecular network construction tool from GNPS [45] ecosystem was used with the following parameters: precursor mass tolerance—0.005 Da, Fragment mass tolerance—0.05 Da, Min Pairs Cos—0.7 Da, Network TopK—10, Minimum matched fragment ions—3, Minimum cluster size—2. Library search parameters: min matched peaks—6, score threshold—0.7.

## 4. Conclusions

Untargeted LC-MS-based studies, especially those dedicated to plants, are always related to various disadvantages due to specifics of the used apparatus and complexity of the object. In the present work, we demonstrated the ways to discard them using the example of a highly important species for the food industry—*Humulus lupulus*. Critical steps in LC-MS data preprocessing were fully described. The approach of combining HCA-based classification and nested feature selection was used for metabolome-based differentiation of brewing cultivars and group-markers compounds search. Obtained labels showed a low correlation with the cultivars’ region of growth but partially agreed with genetic and “usage type” classifications. With the implementation of nested feature selection, the selection bias effect was minimized and an informative feature list was obtained, without annotation-based pre-filtration procedure. Nine compounds that were found to have maximum discriminative power, and multi-step identification including database search and molecular networks construction was performed. Four of them were identified as humulone and its derivatives. Furthermore, isoxanthohumol and structurally similar compounds were found. The markers discovered could be potentially helpful in the development of quality control approaches and further studies oriented to hops metabolome. The experimental design presented in this work could be easily transferred to other types of tasks and objects.

## Figures and Tables

**Table 1:** List of cultivars analyzed in the present study. The last column is dedicated to groups, to which cultivars were assigned by the results of the study.

Cultivar	Region of Growth	Genetic Origin [10]	Type	New Labels
Saaz	Central Europe	European	Aroma	2
Tettnanger		European	Aroma	2
Hallertau Mittelfruh		North American	Aroma	2
Perle		European	Dual use	2
Nugget		North American	Dual use	1
Styrian Cardinal		North American	Dual use	1
Amarillo	North America	North American	Aroma	2
Fuggle		European	Aroma	2
Willamette		European	Aroma	2
Cashmere		North American	Dual use	1
Mosaic		North American	Dual use	1
Cascade		North American	Aroma	2
Kohatu	Oceania	North American	Aroma	2
Wai-iti		European	Aroma	2
Nelson Sauvin		North American	Dual use	1
Galaxy		North American	Dual use	1
Waimea		North American	Dual use	1
Ella		North American	Aroma	1

**Table 2:** List of marker compounds. *m/z* are provided as parent ion values for fragmentation. VIP and fold change values were calculated on the full dataset.

	Compound Name	Retention Time, Min	*m/z*	Ion Type	Predicted Molecular Formula (Error, ppm)	VIP from PLS	Fold Change
1	Isoxanthohumol	10.46	353.1384	[M-H]^−^	C_21_H_22_O_5_ (0.26)	1.73	1.45
2	Unknown	11.4	399.1443	[M-H]^−^	C_22_H_24_O_7_ (3.67)	1.24	1.06
3	Cohumulone	19.37	347.1886	[M-H]^−^	C_20_H_28_O_5_ (0.53)	1.42	2.15
4	Posthumulone	17.43	333.1694	[M-H]^−^	C_19_H_26_O_5_ (0.8)	1.56	2.23
5	Humulone	21.59	361.202	[M-H]^−^	C_21_H_30_O_5_ (1.95)	1.46	0.31
6	Prehumulone	23.2	375.2172	[M-H]^−^	C_22_H_32_O_5_ (0.57)	1.35	2.33
7	Unknown	20.33	385.2371	[M + H]^+^	---	1.31	1.11
8	Unknown	10.01	410.196	[M + H]^+^	---	1.71	0.93
9	Unknown	16.1	371.1811	[M + H]^+^	C_23_H_32_O_4_ (0.67)	1.42	1.03

## Data Availability

Raw data files of the study (both IT-TOF and Orbitrap data) are available at MassIVE: MSV000089747. Used R scripts and the peak table are published at the GitHub repository: https://github.com/mukhomorr/Hopomics (accessed on 1 July 2022).

## Supplementary Materials

The following are available online at https://www.mdpi.com/article/10.3390/metabo12100945/s1, Figure S1: TIC chromatograms of QC sample, Figure S2: XIC chromatograms of marker compounds m/z in QC sample, Figure S3: Part of obtained molecular network describing connectivity between fragmentation spectra of marker compounds, Figure S4: Dendrogram obtained after HCA of samples using only selected features, Figure S5: Plot obtained after PCA of samples using only selected features, Figure S6: Projection of study samples on principal components surface. Newly assigned groups are demonstrated by colour. Sample names description could be found in table S1, Figure S7: Fragmentation spectra of marker compounds. Number corresponds to Table 1 in the main text, Table S1: List of cultivars, analyzed in present study. Last column is dedicated to groups, to which cultivars were assigned by results of the study.
