# Supplementary material for: Hopomics: Humulus lupulus Brewing Cultivars Classification Based on LC-MS Profiling and Nested Feature Selection
Source: Metabolites. 2022 Oct 5;12(10):945. doi: 10.3390/metabo12100945 (PMC9609554; doi:10.3390/metabo12100945)
Supplement: Supplementary file 1 [file metabolites-12-00945-s001.zip › metabolites-1897969-supplementary.pdf]

Supplementary Materials

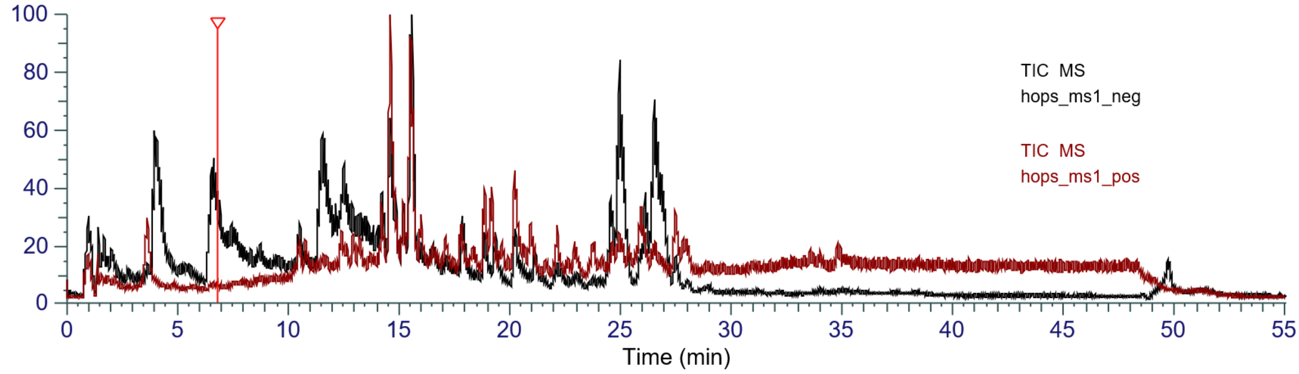

Figure S1. TIC chromatograms of QC sample.

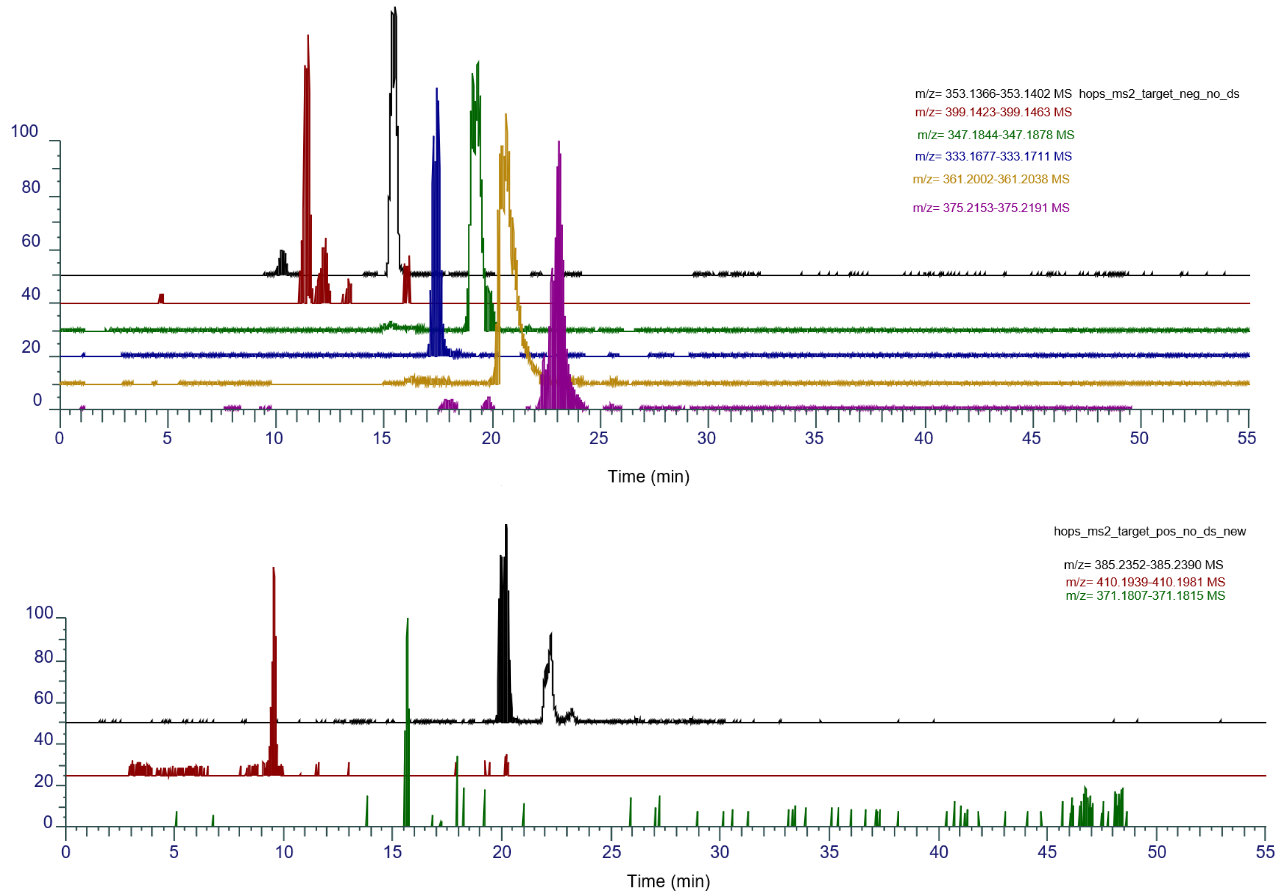

Figure S2. XIC chromatograms of marker compounds m/z in QC sample

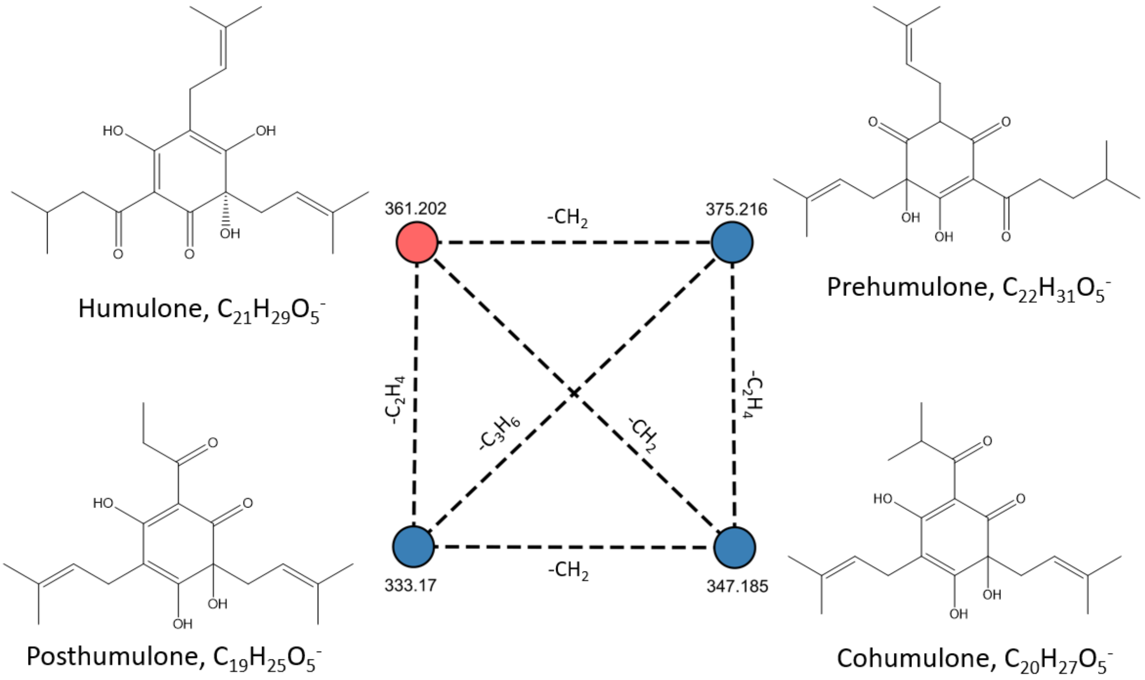

Figure S3. Part of obtained molecular network describing connectivity between fragmentation spectra of marker compounds.

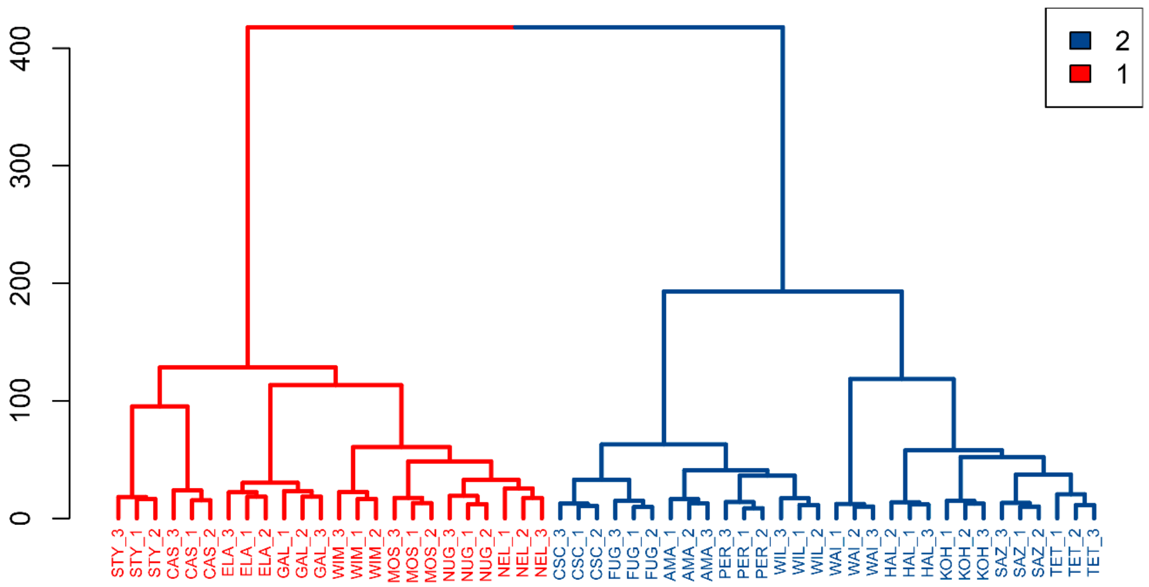

Figure S4. Dendrogram obtained after HCA of samples using only selected features

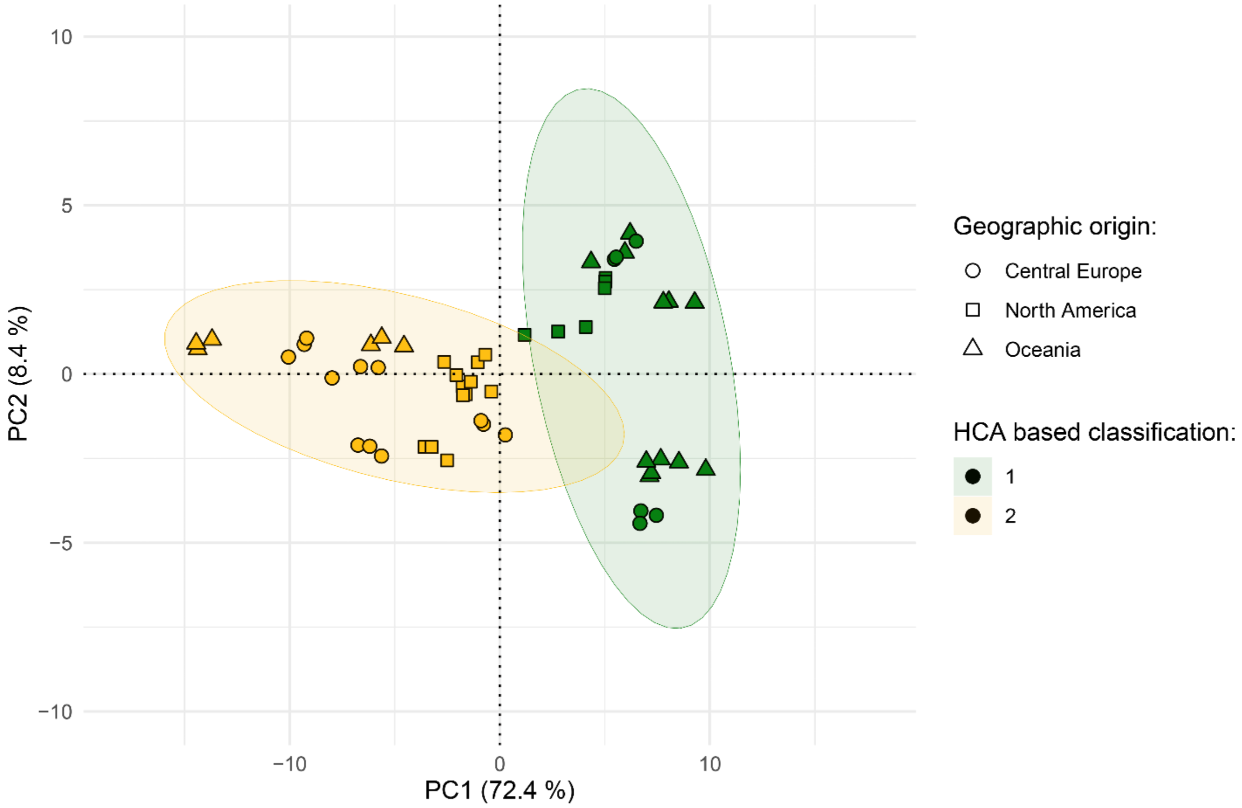

Figure S5. Plot obtained after PCA of samples using only selected features

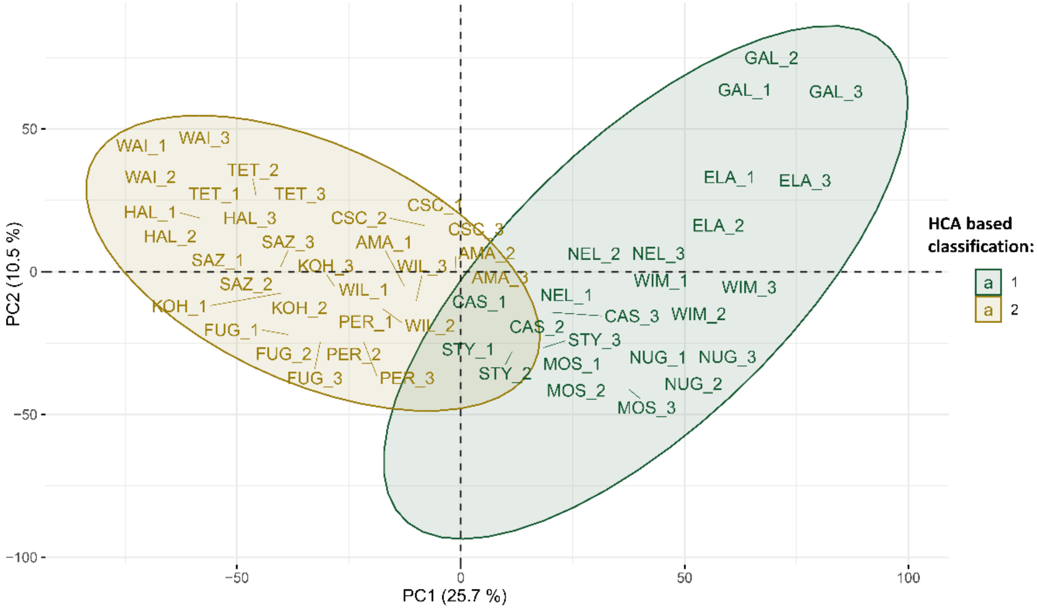

Figure S6. Projection of study samples on principal components surface. Newly assigned groups are demonstrated by colour. Sample names description could be found in table S1.

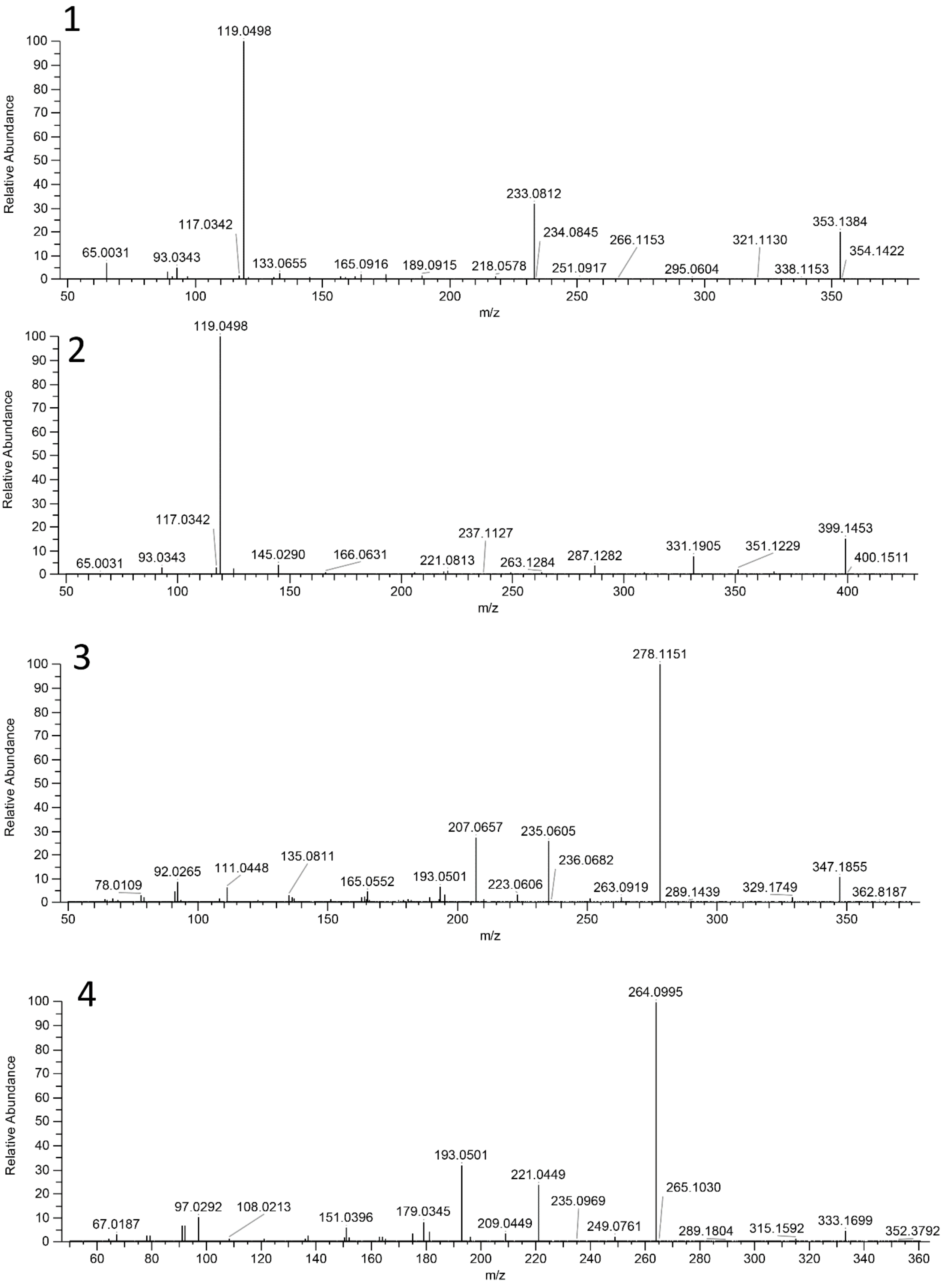

1
119.0498
233.0812
117.0342
234.0845
266.1153
321.1130
353.1384
354.1422
65.0031
93.0343
133.0655
165.0916
189.0915
218.0578
251.0917
295.0604
338.1153
2
119.0498
117.0342
237.1127
399.1453
331.1905
351.1229
65.0031
93.0343
145.0290
166.0631
221.0813
263.1284
287.1282
400.1511
3
278.1151
207.0657
235.0605
236.0682
135.0811
347.1855
78.0109
92.0265
111.0448
165.0552
193.0501
223.0606
263.0919
289.1439
329.1749
362.8187
4
264.0995
193.0501
221.0449
265.1030
235.0969
67.0187
97.0292
108.0213
151.0396
179.0345
209.0449
249.0761
289.1804
315.1592
333.1699
352.3792
Relative Abundance
m/z

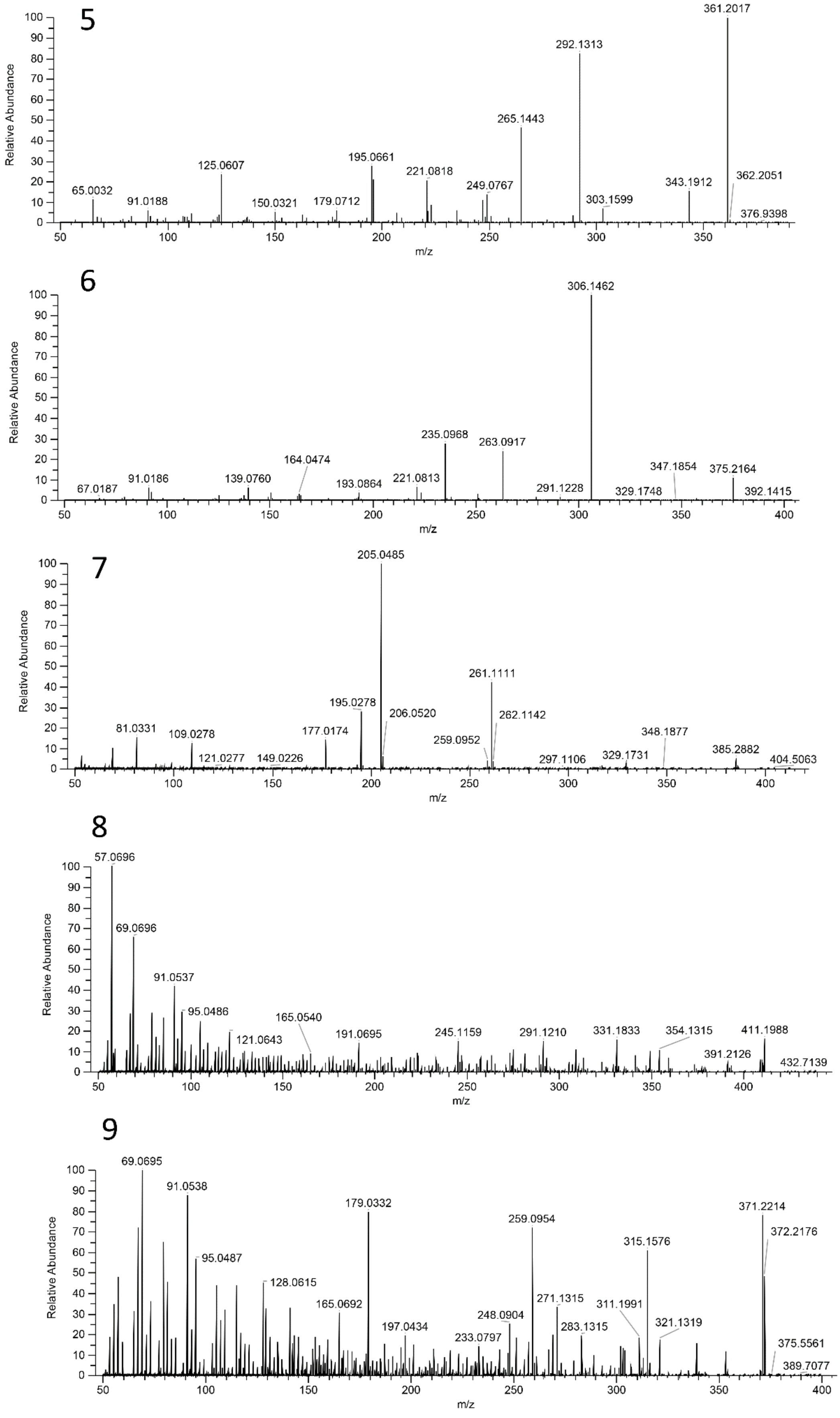

Figure S7. Fragmentation spectra of marker compounds. Number corresponds to Table 1 in the main text

Table S1. List of cultivars, analyzed in present study. Last column is dedicated to groups, to which cultivars were assigned by results of the study.

Cultivar	Sample acronym	Region of growth	Genetic origin [10]	Type	New labels
Saaz	SAZ	Central Europe	European	Aroma	2
Tettnanger	TET		European	Aroma	2
Hallertau Mittelfruh	HAL		North American	Aroma	2
Perle	PER		European	Dual use	2
Nugget	NUG		North American	Dual use	1
Styrian Cardinal	STY		North American	Dual use	1
Amarillo	AMA	North America	North American	Aroma	2
Fuggle	FUG		European	Aroma	2
Willamette	WIL		European	Aroma	2
Cashemere	CAS		North American	Dual use	1
Mosaic	MOS		North American	Dual use	1
Cascade	CSC		North American	Aroma	2
Kohatu	KOH	Oceania	North American	Aroma	2
Wai-iti	WAI		European	Aroma	2
Nelson Sauvin	NEL		North American	Dual use	1
Galaxy	GAL		North American	Dual use	1
Waimea	WIM		North American	Dual use	1
Ella	ELA		North American	Aroma	1
